# Predicting the Blood-Brain Barrier Permeability of New Drug-Like Compounds via HPLC with Various Stationary Phases

**DOI:** 10.3390/molecules25030487

**Published:** 2020-01-23

**Authors:** Małgorzata Janicka, Małgorzata Sztanke, Krzysztof Sztanke

**Affiliations:** 1 Department of Physical Chemistry, Faculty of Chemistry, Institute of Chemical Science, Maria Curie-Skłodowska University, Maria Curie-Skłodowska Sq. 3, 20-031 Lublin, Poland; malgorzata.janicka@poczta.umcs.lublin.pl; 2 Chair and Department of Medical Chemistry, Medical University, 4A Chodźki Street, 20-093 Lublin, Poland; 3 Laboratory of Bioorganic Synthesis and Analysis, Chair and Department of Medical Chemistry, Medical University, 4A Chodźki Street, 20-093 Lublin, Poland

**Keywords:** HPLC, blood-brain barrier permeability, IAM column, Cholester column, ODS column, QSARs, LFERs

## Abstract

The permeation of the blood-brain barrier is a very important consideration for new drug candidate molecules. In this research, the reversed-phase liquid chromatography with different columns (Purosphere RP-18e, IAM.PC.DD2 and Cosmosil Cholester) was used to predict the penetration of the blood-brain barrier by 65 newly-synthesized drug-like compounds. The linear free energy relationships (LFERs) model (log *BB* = *c* + *eE* + *sS* + *aA* + *bB* + *vV*) was established for a training set of 23 congeneric biologically active azole compounds with known experimental log *BB* (*BB* = *C*_blood_/*C*_brain_) values (*R*^2^ = 0.9039). The reliability and predictive potency of the model were confirmed by leave-one-out cross validation as well as leave-50%-out cross validation. Multiple linear regression (MLR) was used to develop the quantitative structure-activity relationships (QSARs) to predict the log *BB* values of compounds that were tested, taking into account the chromatographic lipophilicity (log *k_w_*), polarizability and topological polar surface area. The excellent statistics of the developed MLR equations (*R*^2^ > 0.8 for all columns) showed that it is possible to use the HPLC technique and retention data to produce reliable blood-brain barrier permeability models and to predict the log *BB* values of our pharmaceutically important molecules.

## 1. Introduction

The biological activity of drugs depends primarily on their pharmacokinetics. The expected pharmacological effect of a given drug can be observed if the pharmacokinetic processes provide its high concentration within the range of the receptor. The amount of drug in tissue and the time that remains an effective concentration depend on the fundamental processes that make up the pharmacokinetic phase of the drug’s action, i.e., liberation (L), absorption (A), distribution (D), metabolism (M) and excretion (E). It is extremely difficult to predict the processes mentioned above because all of them are concentration-dependent and connected with the chemical structure of the agent. Since most drugs must pass through at least one cell membrane to provide the desired effect, for the rational design of drugs, it is vitally important to understand and to be able to predict the solute partitioning in the biomembranes. Drugs can cross membranes by passive or active transport [1,2,3]. While active transport is determined by compounds’ affinities for specific transporters and it uses energy, the most common mode for the passage of xenobiotics is passive transport, which depends on physicochemical properties of the compound, such as lipophilicity, size of the molecule, ionization state, as well as the diffusion coefficient through the membrane and the concentration gradient of the compound [4,5].

One of the most important properties of a potential drug is the ability of its molecule to penetrate the blood-brain barrier (BBB). Potential effective agents that are intended to interact with the central nervous system must be able to cross the BBB and satisfactory transport through the blood-brain barrier is an essential prerequisite for a potential drug to affect the central nervous system. However, the agents that act peripherally should not cross the BBB in order to avoid side effects. In both cases, the permeability of the BBB must be known and it should be evaluated at the earliest possible stage of testing. Doing so allows scientists to choose drug candidates that have more selective pharmacologic properties with fewer side effects and lower toxicities [6].

The common measure of the extent permeation of the blood-brain barrier is the ratio of the concentration of the drug in the brain (*C*_brain_) to the concentration of the drug in the blood (*C*_blood_) or in the plasma (*C*_plasma_), which is expressed as the log *BB* (*BB* = *C*_blood_/*C*_brain_) [7,8,9]. Although measurement of the blood-brain barrier penetration in vivo is essential, the procedure is time-consuming, expensive and difficult. In addition, in recent years, an emphasis has been placed on modelling the log *BB* permeation to avoid unethical animal testing. Over the past three decades, various models have been proposed for predicting the BBB permeation and they have suggested different descriptors of the physicochemical properties of substances. Research shows that the penetration of the blood-brain barrier of the compound depends on its hydrogen bonding potential, lipophilicity and size. The BBB penetration is promoted by a weak potential for hydrogen bonding and high lipophilicity [4,5,7,8,9].

As noted, lipophilicity is one of the most important features affecting the biological activity. Octanol-water partition coefficients (logs *P*) are the most extensively used measure of lipophilicity in modelling the biological partition/distribution. This value can be determined by the classical shake-flask method, which is a time-consuming and tedious procedure. Contrarily, the liquid chromatography is a convenient, reliable and efficient method for assessing the partition parameters that describe the lipophilic properties of organic compounds. The background to this is that the same basic molecular interactions determine the behaviour of the solute in both biological and chromatographic systems. Moreover, there is an increasing evidence of the convenience for modelling pharmacokinetic processes chromatographically, especially by reversed-phase liquid chromatography using biomimetic stationary phases. The octadecylsilyl (ODS) stationary phase provides a fast approach, but immobilized artificial membranes (IAMs) are more similar to the membranes of eukaryotic cells and therefore better mimic biological systems [10,11,12]. Artificial membranes are more similar to biological systems because they anchor synthetic phosphatidylcholine analogues to silica [13,14,15,16,17,18]. Cholesterol is one of the major components of many eukaryotic membranes and it seems highly likely that cholesterol immobilized on silica would offer similar possibilities. Currently, the stationary phases with immobilized cholesterol are becoming more and more popular and therefore they are increasingly used to study biological properties of different organic compounds [19,20].

All pharmaceutically relevant compounds [19,21,22,23,24,25,26,27,28,29,30,31,32,33,34,35] were resynthesized in our laboratory for the current research purposes and their structures, belonging to particular groups, are listed in Table 1. They have been shown to possess mainly promising anticancer [19,21,22,23,24,25,26,27,28,29,30,32], analgesic [19,21,31,33], antiviral and antihaemolytic [27] activities. Small molecules **1**–**6** (group I) and **61**–**65** (group VII) are of particular importance as possible anticancer drug candidates for the treatment of human tumours of lung, cervix, breast and ovary [22,23,24]. In addition, the most promising structures **1**–**6** (i.e., showing the minimum embryotoxic concentration higher or comparable to that of aciclovir as well as protective effects on oxidatively-stressed erythrocytes) revealed significant anticancer activities in human tumour cells of pharynx and tongue. The majority of them proved to be more selective than a clinically useful anticancer agent—hydroxycarbamide. Besides, the compound **6** has been shown to possess the remarkable concentration-dependent potency against *Herpes simplex* virus type 1, while revealing a low toxicity to normal Vero cells and inhibiting the oxidatively-induced haemolysis of erythrocytes [27]. In turn, the confirmed remarkable antiproliferative effects of compounds **12**–**17** (group III) may be of benefit in the treatment of human multiple myeloma cells that are susceptible and resistant to thalidomide as well as in human tumour cells of cervix and breast [25,26]. Molecules **1**, **2**, **4**, **5**, **6**, **15**, **19**, **21**, **22**, **24**, **28**, **39**, **63** and **64** have been reported as promising anticancer drug candidates, due to not only their proven significant antiproliferative activities in some human cancer cells but also less toxic effects for normal cells [22,23,24,25,26,27]. Furthermore, test compounds proved to be in vivo active when investigated in the central nervous system. Among analgesic active and relatively low toxic molecules (**8**–**11**, **32**, **34**, **39**, **42**, **48**, **51** and **53**), the structures **8**, **42** and **51** have been shown to produce the strongest antinociceptive effect in mice [19,21,31,33].

## 2. Results 

### 2.1. Chromatographic Results

Retention parameters reported as the log *k* values were calculated by the expression:

(1) $$\log k=\log\frac{\left(t_{R}-t_{0}\right)}{t_{0}}$$

where *t*_R_ and *t*_0_ are the retention times of the solute and a non-retained compound (citric acid), respectively. They were used to calculate the log *k*_w_ values, i.e., logarithms of retention parameter in the buffer as the mobile phase. For this purpose the Soczewiński-Wachtmeister’s equation was used [36]:

log *k* = log *k*_w_ − *sφ* (2)

where *φ* is the volume fraction of organic modifier in the mobile phase; *k* and *k*_w_ are retention parameters corresponding to mixed effluent and buffer as the mobile phase, respectively. The slope *s* is characteristic of a given solute and chromatographic system. Strong linear relationships between log *k* and *φ* values were found for all the compounds in the range of effluent composition examined (*R*^2^ > 0.9). The log *k*_w_ and *s* values obtained from particular chromatographic systems are presented in Table 2. The log *k*_w_ values determined for the ODS (log *k*_w, ODS_), IAM (log *k*_w, IAM_) and Cholester (log *k*_w_, _Cholester_) columns were intercorrelated and the following linear relationships with a very good statistical quality were obtained:

log *k*_w, ODS_ = 0.691(± 0.094) + 0.982(± 0.044) log *k*_w, IAM_ (3)

SD = 0.257; *R*^2^ = 0.8880; *N* = 65

log *k*_w, ODS_ = 0.911(± 0.062) + 0.765(± 0.025) log *k*_w, Cholester_ (4)

SD = 0.192; *R*^2^ = 0.9371; *N* = 65

log *k*_w, IAM_ = 0.356(± 0.073) + 0.721(± 0.029) log *k*_w, Cholester_ (5)

SD = 0.224; *R*^2^ = 0.9068; *N* = 65

Moreover highly significant linear relationships were obtained between log *k*_w_ and *s* values (intercepts and slopes of Equation (2)):

for the ODS column:

log *k*_w, ODS_ = −1.214(± 0.062) + 0.823(± 0.013) *s*_ODS_ (6)

SD = 0.094; *R*^2^ = 0.9850; *N* = 65

for the IAM column:

log *k*_w, IAM_ = −0.779(± 0.026) + 0.638(± 0.006) *s*_IAM_ (7)

SD = 0.052; *R*^2^ = 0.9950; *N* = 65

and for the Cholester column:

log *k*_w, Cholester_ = −0.892(± 0.040) + 0. 798(± 0.010) *s*_Cholester_ (8)

SD = 0.092; *R*^2^ = 0.9910; *N* = 65

### 2.2. Establishment of the LFER Model

The property of the substance can be predicted on the basis of the linear free-energy relationships (LFER), but to do so, the relationship between the chemical structure and the desired property should be identified [37]. A symbolic representation of LFERs model is the equation originally employed by Abraham et al. [38,39,40,41,42]:

SP = *c* + *eE* + *sS* + *aA* + *bB* + *vV* (9)

Here SP is a set of solute properties in a given system, e.g., log *BB* values. The independent values are solute descriptors: *E* is an excess molar refraction, *S* is the dipolarity/polarizability, *A* and *B* are the hydrogen bond acidity (donating ability) and basicity (accepting ability), respectively and *V* is the solute McGowan volume (cm^3^∙mol^−1^/100). Coefficients *c*, *e*, *s*, *a*, *b* and *v* are characteristic for a given biphasic system and solute.

In this study, this equation was established for a training set of 23 azole compounds that were congeneric with those tested in our investigations. For these compounds, we obtained from the literature [43] the experimental log *BB* (*BB* = *C*_brain_/*C*_plasma_) values for rats (Table 3). The actual values of log *BB* ranged from −0.82 to 0.58. The following MLR equation was obtained:

log *BB*_exp._ = 0.934(± 0.166) + 0.191(± 0.107) *E* − 0.605(± 0.134) *S* − 0.743(± 0.137) *A* − 0.768(± 0.177) *B* + 0.545(± 0.104) *V* (10)

*N* = 23; SD = 0.134; *R*^2^ = 0.9039; *F* = 32; *p* < *10^−5^*

**Figure 1:**
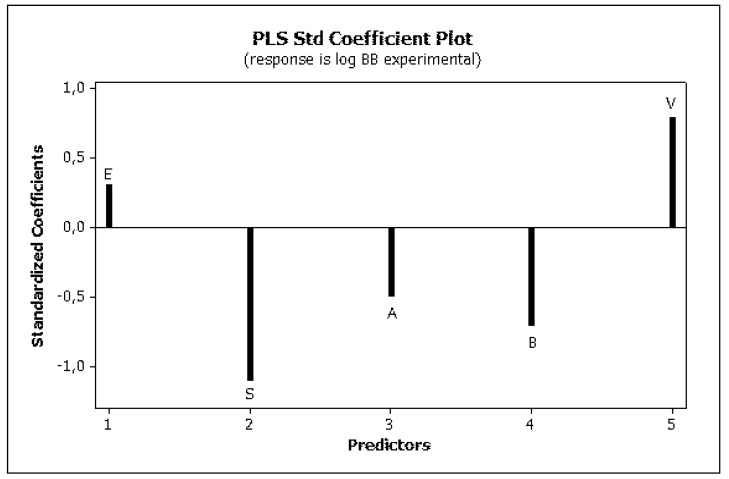
PLS standardized coefficient plot (Equation (10)).

**Figure 2:**
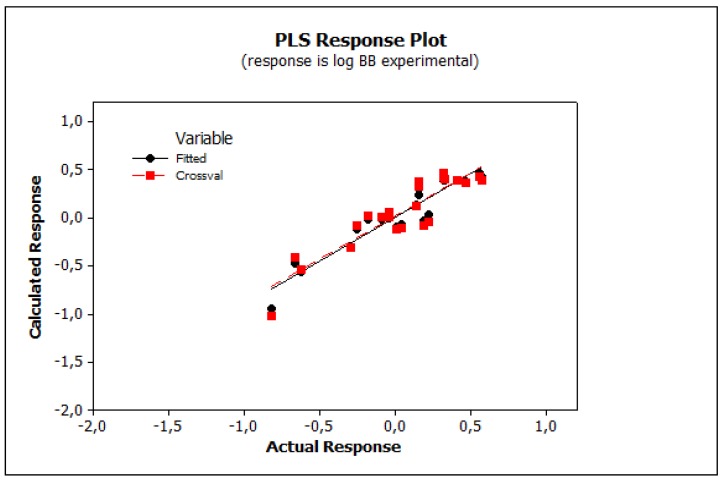
PLS response plot (Equation (10)).

**Figure 3:**
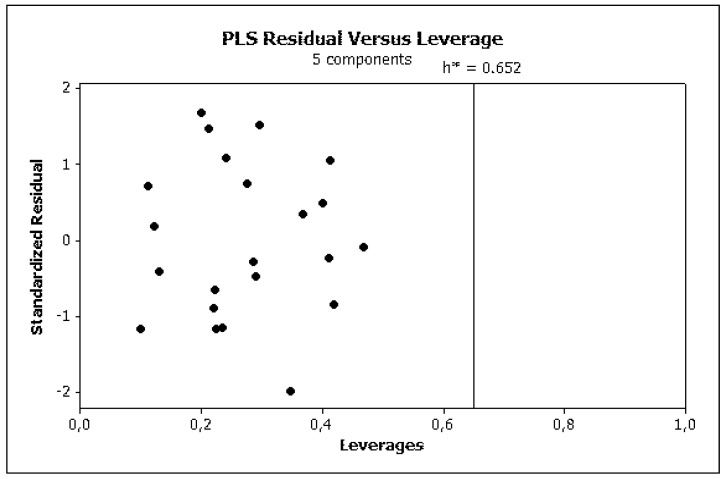
PLS residual vs. leverage (Equation (10)).

Irrelevant cross correlations between the descriptors were observed; the values of *R*^2^ between pairs of descriptors were *E*/*S* 0.66, *E*/*A* 0.01, *E*/*B* 0.25, *E*/*V* 0.60, *S*/*A* 0.06, *S*/*B* 0.69, *S*/*V* 0.72, *A*/*B* 0.20, *A*/*V* 0.05 and *B*/*V* 0.49. This is an important information indicating that there are no significant inter-correlations of structural descriptors, i.e., *E*, *S*, *A*, *B* and *V*. On this basis, we can conclude that non-physical factors do not affect the parameters of Equation (10). The reliability and predictive potency of the model expressed by Equation (10) were estimated by leave-one-out (LOO) cross validation and the parameters that were obtained are presented in Table 4. Figure 1 shows the standardized coefficients for particular descriptors and it confirms the well-known qualitative relationships: compound polarity, i.e., dipolarity/polarizability, hydrogen bond acidity and hydrogen bond basicity expressed by *S*, *A* and *B* values, respectively, decreases the BBB permeation, while the compound size measured by the McGovan volume (*V*) as well as the excess molar refraction *E* (in a minor degree) contribute to increase of log *BB* values [41]. The PLS response plot is presented in Figure 2, which shows the linear regression between the predicted (calculated response) and experimental log *BB* values of the 23 compounds from Table 3. The residual versus leverage plot in Figure 3 proves that the model that was obtained is valid for the domain in which it was developed [44]. The warning leverage limit (h*) was calculated according to:

(11) $$h^{*}=\frac{3m}{n}$$

where *m* is the number of descriptors and *n* is the number of observations (compounds) in the dataset.

Equation (10) was used to calculate the log *BB* values for our 65 pharmaceutically relevant compounds (Table 5).

### 2.3. Establishment of QSARs Models

Efforts to predict the biological activity (including the BBB permeation) based on the properties of substances led to the development of Quantitative Structure Activity Relationships (QSARs) and Quantitative Retention Activity Relationships (QRARs) methodology [37,44,45,46,47,48]. Various models and approaches have been developed to predict the penetration of the blood-brain barrier based on various physicochemical properties of molecules, including the lipophilicity, molecular size, polarizability, polar surface area and the number of groups that can establish potential hydrogen bonds [49,50,51,52,53,54,55,56,57,58,59,60,61,62,63]. It is reasonable to assume that the combination of theoretical and experimental data increases the reliability of the anticipated transport of the drug across the blood-brain barrier [64,65,66]. The chromatographic retention parameter is one of the most popular experimental values used to characterize the properties (lipophilicity/hydrophobicity) of compounds used in QRAR and QSAR studies. The solute retention depends on the changes in free energy that are associated with the distribution of the solute between the mobile and stationary phases in a given chromatographic system. Thus, it is possible to use the values obtained on HPLC columns that imitate biomembranes for modelling the blood-brain barrier permeation.

In our investigations in which we modelled the blood-brain permeation of 65 biologically active molecules, the chromatographic lipophilicity (log *k_w_*) (Table 2), polarizability (*α*) and topological polar surface area (TPSA) (Table 5) were considered. The following MLR equations corresponding to various stationary phases were obtained:

for ODS column:

log *BB* = 0.587(± 0.127) + 0.011(± 0.020) log *k*_w. ODS_ − 0.013(± 0.001) TPSA + 0.008(± 0.004) *α* (12)

*N* = 65; SD = 0.069; *R*^2^ = 0.8474; *F* = 113; *p* < 10^−6^; VIF < 3.1

for IAM column:

log *BB* = 0.578(± 0.127) + 0.007(± 0.019) log *k*_w, IAM_ − 0.013(± 0.001) TPSA + 0.009(± 0.004) *α* (13)

*N* = 65; SD = 0.069; *R*^2^ = 0.8469; *F* = 113; *p* < 10^−6^; VIF < 2.7

and for Cholester column:

log *BB* = 0.595(± 0.139) + 0.008(± 0.017) log *k*_w, Cholester_ − 0.013(± 0.001) TPSA + 0.009(± 0.004) *α* (14)

*N* = 65; SD = 0.069; *R*^2^ = 0.8471; *F* = 113; *p* < 10^−6^; VIF < 3.7

**Figure 4:**
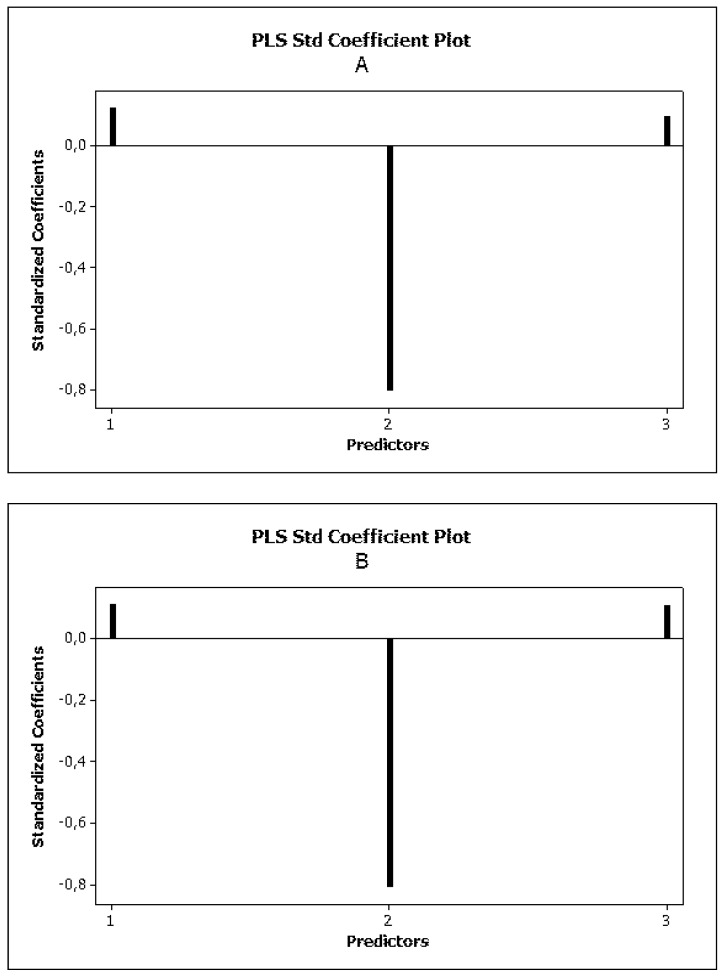

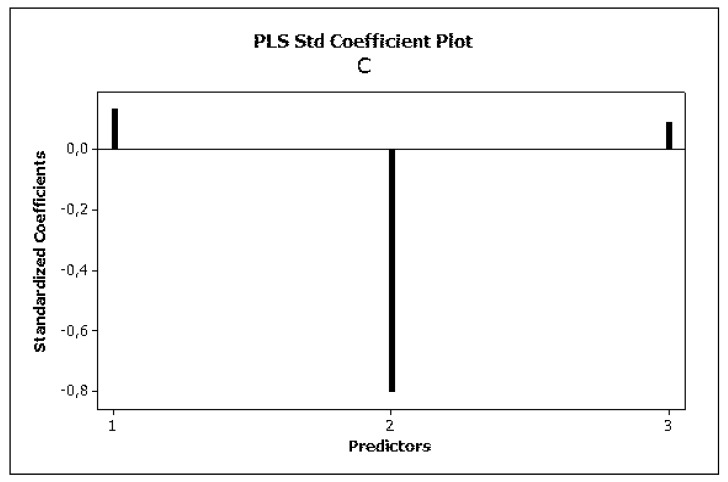
PLS standardized coefficient plots obtained for Equation (12) (**A**), Equation (13) (**B**) and Equation (14) (**C**).

The statistics of Equations (12)–(14) were very good, i.e., *R*^2^ > 0.8 for all chromatographic systems and all variance inflation factors (VIF < 5) indicated that the variables were correlated moderately. The reliability of the MLR models, as expressed by Equations (12)–(14), were estimated by leave-one-out as well as leave-50%-out cross validation (Table 4). In each case, the log *k_w_* and *α* values provided positive inputs to the log *BB*, while TPSA decreased the permeation of the blood-brain barrier (Figure 4). 

**Figure 5:**
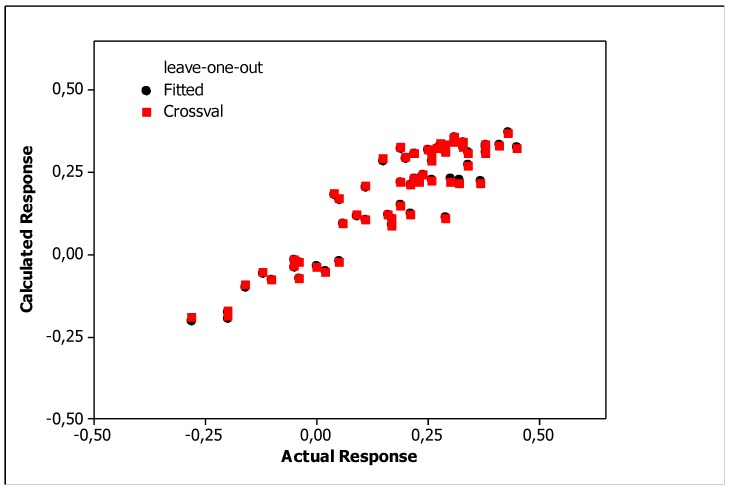

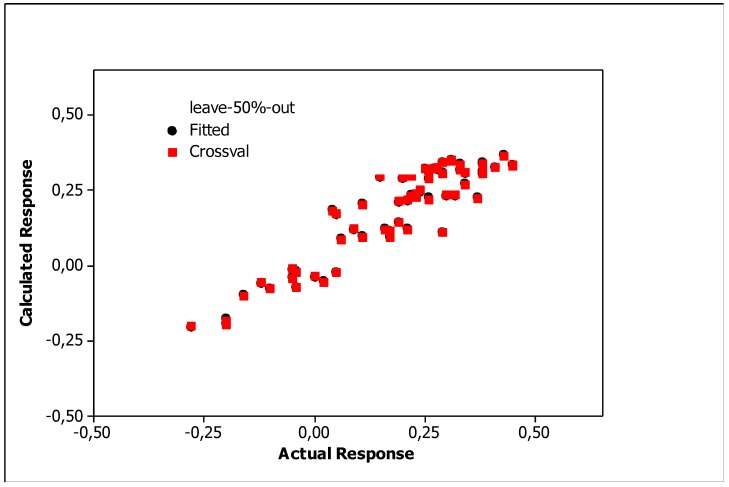
PLS response plots obtained for Equation (12).

**Figure 6:**
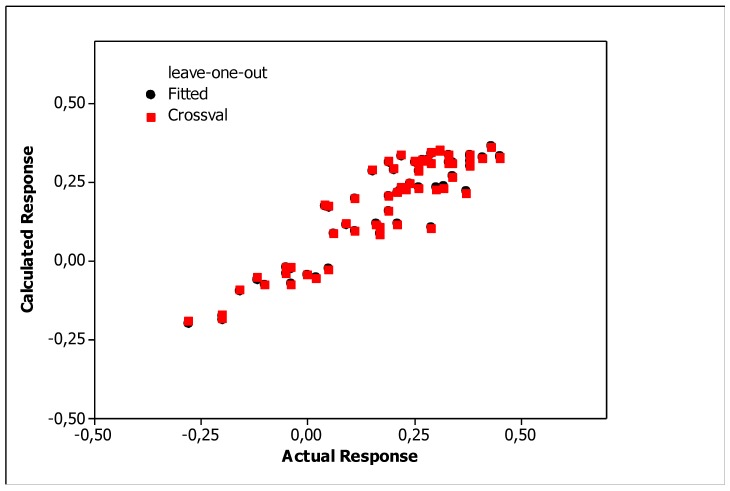

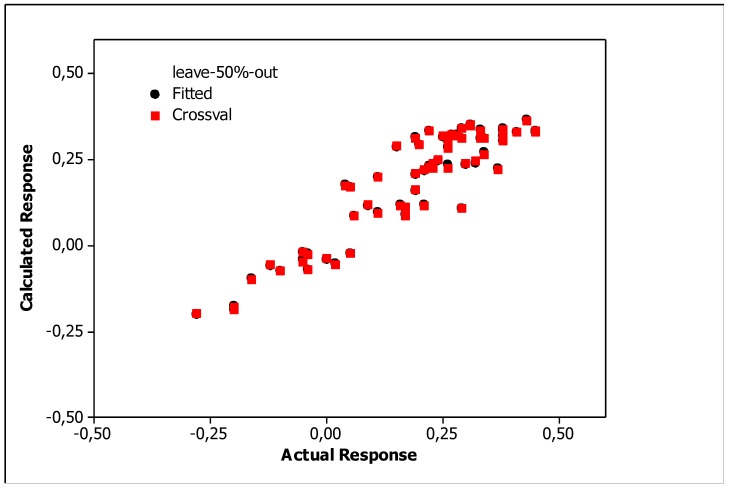
PLS response plots obtained for Equation (13).

**Figure 7:**
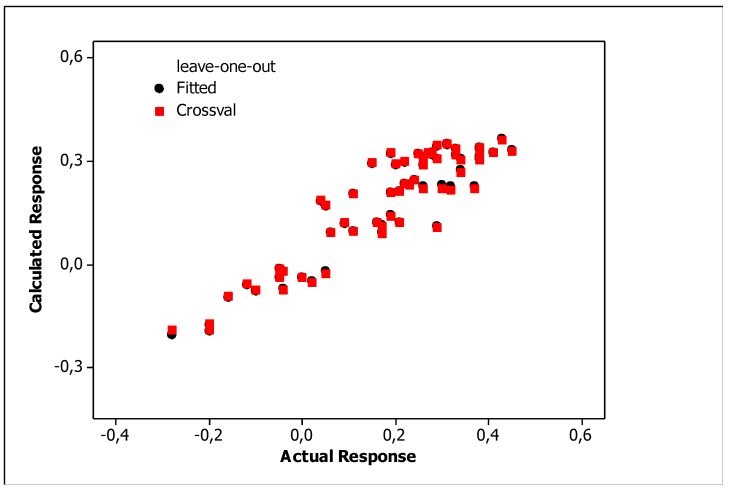

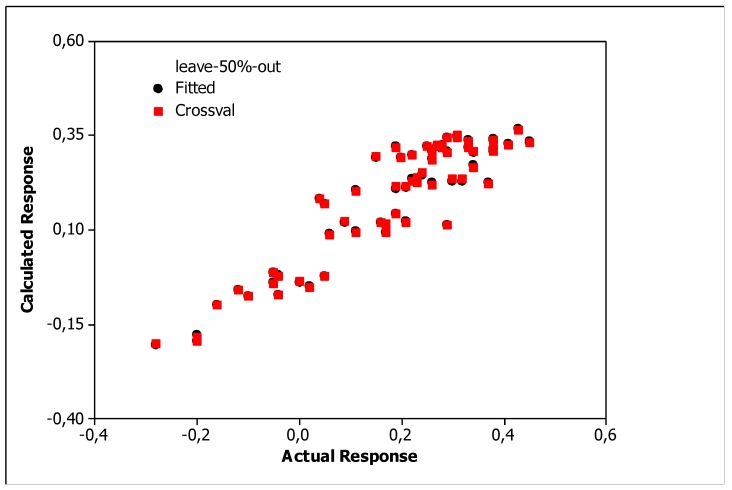
PLS response plots obtained for Equation (14).

The relationships showed that molecular polarizability and lipophilicity promote increases in the log *BB*, while polar surface area decreased the ability of a substance to cross the BBB. The correlations between the log *BB* values calculated according to the LFER model (Equation (10)) and the optimized QSARs models, i.e., by Equations (12)–(14) are presented in Figure 5, Figure 6 and Figure 7, which shows the response plots obtained by PLS for particular stationary phases.

**Figure 8:**
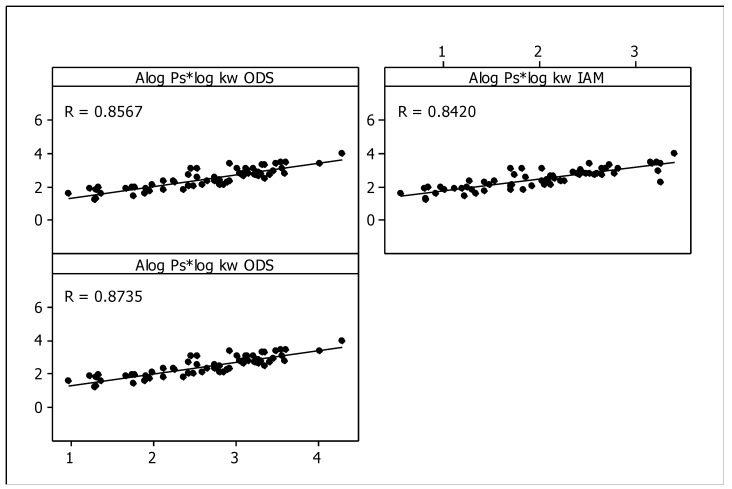
Alog *P*_s_ vs. log *k*_w_ relationships obtained for ODS, IAM and Cholester columns.

To assess the significance of chromatographic parameters, the lipophilicity descriptors, log *k*_w_ were compared with partition coefficients in the *n*-octanol-water system, i.e., the log *P* values. The relationships between log *P* values calculated from molecular structures of the tested compounds and obtained by use of Alog *P*_s_ algorithm [67,68] and their chromatographic factors (i.e., log *k*_w, ODS_, log *k*_w_, _IAM_ and log *k*_w, Cholester_, respectively) were established. Good linear correlations between these descriptors (*R* > 0.8), that are observed in Figure 8, confirmed that chromatographic parameters can be used as lipophilicity descriptors in case of the studied compounds (Figure 8).

Moreover the following QSAR equation including Alog *P*_s_, TPSA and *α* descriptors was established:

log *BB* = 0.627(± 0.115) + 0.077(± 0.028) Alog *P*_s_ − 0.012(± 0.001) *TPSA* + 0.001(± 0.004) *α* (15)

*N* = 65; SD = 0.065; *R*^2^ = 0.8639; *F* = 130; *p* < 10^−6^; VIF < 4.3; *Q^2^* = 0.8572; MSE = 0.004287; MSEcv^*^ = 0.004218; MSEcv^**^ = 0.004218; PRESS^*^ = 0.2900; PRESS^**^ = 0.2698.

The statistics of Equation (15) proved to be very good and similar to those obtained for Equations (12)–(14), which confirms their ability in predicting the blood brain barrier permeation.

## 3. Materials and Methods

### 3.1. Reagents

Acetonitrile (HPLC grade) was purchased from Merck (Lublin, Poland). Citric acid and Na_2_HPO_4_ (both pure) were supplied from POCh (Lublin, Poland). Distilled water was obtained from Direct-Q3 UV apparatus (Millipore, Warsaw, Poland).

### 3.2. Instrumental

Shimadzu Vp (Shimadzu, Izabelin, Poland) liquid chromatographic system equipped with LC 10AT pump, SPD 10A UV-Vis detector, SCL 10A system controller, CTO-10 AS chromatographic oven and Rheodyne injector valve with a 20 µL loop was applied in HPLC measurements. Three stationary phases were employed:

- Purosphere RP-18e (ODS), 125 × 4 mm i.d., 5 µm (Merck);
- IAM.PC.DD2 100 × 4.6 mm i.d., 10 µm (Regis Chemicals Company, Morton Grove, IL, USA);
- Cosmosil Cholester, 75 × 2 mm i.d., 2.5 µm (Genore, Warsaw, Poland).

### 3.3. Chromatographic Conditions and Test Substances

As mobile phases buffer-acetonitrile mixtures were used. The buffer was prepared from 0.01 mol L^−1^ solutions of Na_2_HPO_4_ and citric acid and the pH 7.4 value was fixed before mixing with organic modifier. With the ODS column acetonitrile concentration in the effluent, expressed as a volume fraction (*φ*, *v*/*v*), was changed in the range 0.3–0.6, with the constant step of 0.1. The flow rate was 1 mL min^−1^. With the IAM column acetonitrile concentration was changed in the range 0.2–0.5, also with the constant step of 0.1 and the flow rate was 1.3 mL min^−1^. With the Cosmosil Cholester column acetonitrile concentration was changed in the range 0.4–0.6, with the constant step of 0.05 and the flow rate was 0.4 mL min^−1^. Samples of test compounds were dissolved in acetonitrile—c.a. 0.005 mg mL^−1^. All the compounds proved to be in the neutral form in solution under experimental conditions and had values of peak asymmetry factor in the acceptable range. They were detected under UV light at 210 and 254 nm. All measurements were carried out at 25 °C. The dead time values were measured from non-retained compound (citric acid) peaks. All reported log *k_w_* values are the average of at least three independent measurements. The extrapolated retention coefficients (logs *k*_w_) achieved by HPLC on ODS, IAM and Cholester stationary phases were used for modelling the log *BB* permeation of 65 drug-like compounds employed as a whole test set (Table 1).

### 3.4. In silico Calculations

Molecular descriptors (*E*, *S*, *A*, *B* and *V*), molecular weight (MW), topological polar surface area (TPSA) and polarizability (*α*) of compounds were evaluated by ACD/Percepta software (Łodź, Poland).

### 3.5. Statistical Analysis

Linear regression, multiple linear regression (MLR), partial last square (PLS), leave-one-out (LOO) and leave-50%-out cross validation were performed using Minitab 16 software (Minitab Inc., State College, PA, USA).

## 4. Discussion

The usefulness of HPLC with three different reverse-phase columns (including two imitating biosystems) for predicting the blood-brain barrier (BBB) permeability of 65 structurally related drug-like molecules was highlighted in our investigations. QSAR models predicting the BBB permeation were built on the basis of experimentally accessible log *k*_w_ values of the test molecules together with their important in silico molecular descriptors. The obtained results confirmed that all three stationary phases, i.e., octadecylsilane, immobilized artificial membrane and cholesterol immobilized on silica gel analogously described the lipophilic properties of the studied solutes (Equations (3)–(5)).

In our studies the extrapolated retention parameters (logs *k*_w_) were used as they are preferred in QSARs instead of the isocratic log *k* values and usually employed in correlation studies with in silico molecular descriptors and drug-likeness properties in case of pharmaceutics and drug-like molecules. It should be noted that chromatographically derived retention parameters are very useful descriptors in QSAR modelling as the partitioning process between the stationary and mobile phase of a solute investigated mimics a membrane penetration process of a pharmaceutic or potential drug candidate [19,21,33,37].

Highly significant linear relationships were obtained between intercepts and slopes of Equation (2), i.e., the log *k*_w_ and *s* values, for particular reversed-phase stationary phases. These correlations confirmed not only the congenereity between compounds that were investigated, but also suggested that the log *k*_w_ and *s* values may be considered as alternative lipophilicity descriptors [19,21,37] in case of structurally related small molecules that were tested. In our studies compounds bearing more hydrophobic substituents revealed the greater *s* values. This observation is consistent with other research findings showing that more hydrophobic molecules reveal greater slopes [21]. According to the background retention theory the *s* values are related to the solute/mobile phase and the solvent/stationary phase net interactions [21,36,37].

The obtained results showed that the chromatographic retention parameters obtained using three stationary phases recruited as well as important in silico molecular descriptors can be recommended to derive the reliable QSAR models for predicting the blood-brain barrier permeability in case of our structurally related small molecules, considered as a test set of potential drug candidates.

The calculated log *P* values were obtained by using Alog *P*_s_ algorithm and compared with the experimental log *k*_w, ODS_, log *k*_w_, _IAM_ and log *k*_w, Cholester_ values, respectively. This estimation was essential to check the validity of the obtained results through correlation with the log *BB* values. 

## 5. Conclusions

Experimental literature data of the log *BB* (for rats) for a training set of 23 biologically active compounds (including drugs) were correlated against five solute descriptors (*E*, *S*, *A*, *B* and *V*) and the established equation (Equation (10)) was validated. The standardized coefficients obtained for particular descriptors confirmed that the compound polarity, i.e., dipolarity/polarizability (*S*), hydrogen bond acidity (*A*) and hydrogen bond basicity (*B*) decreases the blood-brain barrier (BBB) permeation while the compound size measured by the McGovan volume (*V*) and the excess molar refraction (*E*) contribute to increase in the log *BB* values. The Equation (10) was used to calculate the log *BB* values for 65 newly synthesized compounds being considered as potential drugs. 

The blood-brain barrier permeability of the compounds that were tested was modelled by three descriptors, i.e., the chromatographic lipophilicity (log *k*_w_), polarizability (*α*) and topological polar surface area (TPSA). Using a simple statistical model (i.e., MLR), three structure-activity relationships were obtained for each chromatographic system on endcapped octadecylsilyl, immobilized artificial membrane and cholesteryl stationary phases (Equations (12)–(14)). The log *BB* values calculated according to Equation (10) and predicted from Equations (12)–(14) were compared and highly significant relationships were obtained between them. The relationships were confirmed by leave-one-out as well as leave-50%-out cross validation, implying that the models are robust and reliable. The results that were obtained showed that, in the case of the compounds that were studied (65 weak organic bases), each of the stationary phases used in the chromatographic measurements was equally useful. From practical and economic perspectives, the Cholester microcolumn is recommended because it allows the acquisition of more data in a shorter time with lower costs. 

We showed that it is possible to use the HPLC technique to build a reliable model for predicting which our organic compounds (congeneric in structure to the training set of azole molecules) can penetrate the blood-brain barrier in rats. The investigations highlighted the key role of chromatographic techniques and QSARs methods in reducing unethical animal testing.

The presented results will be particularly useful in further more extensive in vivo research, which is planned to be carried out on our small molecules considered as potential drugs.

## Figures and Tables

**Table 1:** Compounds tested.

Group	Compound No.; R, R’	Chemical Name	References
Group I 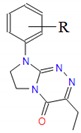	**1**: R = H **2**: R = 4-CH_3_ **3**: R = 2-Cl **4**: R = 3-Cl **5**: R = 4-Cl **6**: R = 3,4-Cl_2_	8-(*R*-phenyl)-3-ethyl-7,8-dihydroimidazo[2,1-*c*][1,2,4]triazin-4(6*H*)-ones	[22,24,27]
Group II 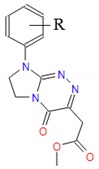	**7**: R = H **8**: R = 4-CH_3_ **9**: R = 4-OCH_3_ **10**: R = 4-OC_2_H_5_ **11**: R = 4-Cl	Methyl [4-oxo-8-(*R*-phenyl)-4,6,7,8-tetrahydroimidazo[2,1-*c*][1,2,4]triazin-3-yl]acetates	[21]
Group III 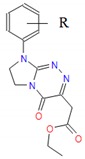	**12**: R = H **13**: R = 4-CH_3_ **14**: R = 4-OCH_3_ **15**: R = 3-Cl **16**: R = 4-Cl **17**: R = 3,4-Cl_2_	Ethyl [4-oxo-8-(*R*-phenyl)-4,6,7,8-tetrahydroimidazo[2,1-*c*][1,2,4]triazin-3-yl]acetates	[25,26]
Group IV 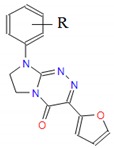	**18**: R = H **19**: R = 2-CH_3_ **20**: R = 4-CH_3_ **21**: R = 2,3(-CH_3_)_2_ **22**: R = 2-OCH_3_ **23**: R = 4-OCH_3_ **24**: R = 2-Cl **25**: R = 3-Cl **26**: R = 4-Cl **27**: R = 3,4-Cl_2_ **28**: R = 2,6-Cl_2_	8-(*R*-phenyl)-3-(2-furanyl)-7,8-dihydroimidazo[2,1-*c*][1,2,4]triazin-4(6*H*)-ones	[19,28,29,30]
Group V 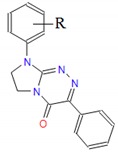	**29**: R = H **30**: R = 2-CH_3_ **31**: R = 3-CH_3_ **32**: R = 4-CH_3_ **33**: R = 2-OCH_3_ **34**: R = 4-OCH_3_ **35**: R = 4-OC_2_H_5_ **36**: R = 2,3(-CH_3_)_2_ **37**: R = 2-Cl **38**: R = 3-Cl **39**: R = 4-Cl **40**: R = 3,4-Cl_2_	8-(*R*-phenyl)-3-phenyl-7,8-dihydroimidazo[2,1-*c*][1,2,4]triazin-4(6*H*)-ones	[19,31,32,33]
Group VI 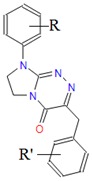	**41**: R = H; R’ = H **42**: R = H; R’ = 2-Cl **43**: R = H; R’ = 3-Cl **44**: R = H; R’ = 4-Cl **45**: R = 4-CH_3_; R’ = H **46**: R = 4-CH_3_; R’ = 4-CH_3_ **47**: R = 4-CH_3_; R’ = 3-CH_3_ **48**: R = 4-CH_3_; R’ = 2-Cl **49**: R = 4-CH_3_; R’ = 3-Cl **50**: R = 4-CH_3_; R’ = 4-Cl **51**: R = 4-OC_2_H_5_; R’ = H **52**: R = 4-OC_2_H_5_; R’ = 4-CH_3_ **53**: R = 4-OC_2_H_5_; R’ = 2-Cl **54**: R = 4-OC_2_H_5_; R’ = 3-Cl **55**: R = 4-OC_2_H_5_; R’ = 4-Cl **56**: R = 2-CH_3_; R’ = 2-Cl **57**: R = 4-Cl; R’ = H **58**: R = 4-Cl; R’ = 2-Cl **59**: R = 4-Cl; R’ = 3-Cl **60**: R = 4-Cl; R’ = 4-Cl	8-(*R*-phenyl)-3-benzyl/3-(*R*’-benzyl)-7,8-dihydroimidazo[2,1-*c*][1,2,4]triazin-4(6*H*)-ones	[19,21]
Group VII 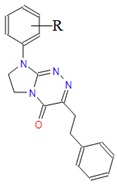	**61**: R = H **62**: R = 4-CH_3_ **63**: R = 2-Cl **64**: R = 4-Cl **65**: R = 3,4-Cl_2_	8-(*R*-phenyl)-3-(2-phenylethyl)-7,8-dihydroimidazo[2,1-*c*][1,2,4]triazin-4(6*H*)-ones	[22,23]

**Table 2:** Parameters of the Soczewiński-Wachtmeister’s equation obtained for various columns.

Compound Tested	ODS		IAM		Cholester	
	log *k*_w, ODS_	*s*_ODS_	log *k*_w, IAM_	*s*_IAM_	log *k*_w, Cholester_	*s*_Cholester_
**1**	0.97	2.70	0.55	2.10	0.20	1.25
**2**	1.30	3.12	1.00	2.88	0.57	1.84
**3**	1.22	3.02	0.80	2.51	0.46	1.70
**4**	1.33	3.15	0.84	2.60	0.62	1.82
**5**	1.78	3.56	1.24	3.25	1.18	2.61
**6**	2.53	4.49	1.85	4.11	2.05	3.61
**7**	1.28	3.09	0.81	2.38	0.58	1.92
**8**	1.90	3.82	1.33	3.33	1.29	2.75
**9**	1.30	3.02	0.81	2.45	0.57	1.82
**10**	1.95	3.91	1.42	3.39	1.36	2.81
**11**	2.36	4.44	1.82	4.39	1.82	3.55
**12**	1.75	3.61	1.21	3.11	1.11	2.55
**13**	2.37	4.36	1.70	3.85	1.92	3.58
**14**	1.37	3.15	0.91	2.58	0.65	1.92
**15**	2.85	4.90	2.05	4.45	2.41	4.15
**16**	2.80	4.88	2.11	4.51	2.37	4.19
**17**	3.16	5.33	2.42	5.01	2.77	4.61
**18**	2.12	4.06	1.29	3.23	1.43	3.12
**19**	1.74	3.48	0.96	2.72	1.20	2.78
**20**	2.43	4.39	1.69	3.90	2.03	3.68
**21**	2.26	4.20	1.42	3.51	1.66	3.33
**22**	1.67	3.48	1.11	3.02	1.22	2.68
**23**	1.91	3.82	1.19	3.13	1.53	3.05
**24**	2.25	4.22	1.27	3.25	1.34	2.95
**25**	2.75	4.85	2.08	4.55	2.36	4.05
**26**	2.65	4.59	2.02	4.44	2.29	4.15
**27**	3.46	5.68	3.23	6.30	2.92	4.85
**28**	2.53	4.45	1.70	3.90	2.05	3.66
**29**	2.49	4.51	1.91	4.22	2.34	4.02
**30**	2.12	4.01	1.53	3.52	1.88	3.50
**31**	2.93	5.02	2.26	4.68	2.77	4.42
**32**	2.93	5.06	2.22	4.81	2.86	4.65
**33**	1.98	3.99	1.47	3.62	1.69	2.94
**34**	2.59	4.62	1.71	3.91	2.27	3.80
**35**	3.09	5.33	2.14	4.48	2.80	4.61
**36**	2.74	4.85	1.85	4.05	2.34	3.60
**37**	2.43	4.60	1.73	3.84	1.96	3.65
**38**	3.29	5.70	2.64	5.41	3.20	5.20
**39**	3.41	5.20	2.57	5.32	3.19	5.30
**40**	3.48	5.90	3.26	6.40	3.86	6.02
**41**	2.90	5.02	3.26	6.45	2.23	3.75
**42**	3.28	5.10	2.34	4.78	2.83	4.71
**43**	3.59	5.85	2.48	5.18	2.90	4.80
**44**	3.60	5.62	2.39	4.81	3.01	4.70
**45**	3.35	5.55	2.15	4.69	2.74	4.59
**46**	3.04	5.30	2.51	5.22	3.15	5.05
**47**	3.09	5.24	2.59	5.14	3.09	5.06
**48**	3.12	5.11	2.65	5.21	3.24	4.90
**49**	3.31	5.41	2.78	5.50	3.40	5.33
**50**	3.22	5.55	2.81	5.62	3.39	5.34
**51**	3.28	5.61	2.11	4.51	2.68	4.55
**52**	3.14	5.36	2.43	5.02	3.07	5.02
**53**	2.93	5.02	2.51	5.17	3.21	5.15
**54**	3.35	5.55	2.73	5.61	3.31	5.22
**55**	3.32	5.66	2.72	5.44	3.36	5.35
**56**	3.01	4.95	2.02	4.31	2.34	4.05
**57**	3.11	5.05	2.52	5.06	2.98	4.85
**58**	3.55	5.65	3.22	6.32	3.74	5.62
**59**	3.61	5.85	3.15	6.01	3.67	5.75
**60**	4.02	6.45	3.17	6.18	3.68	5.82
**61**	2.80	4.92	2.07	4.45	2.33	4.02
**62**	3.23	5.44	2.41	5.01	2.84	4.70
**63**	2.46	4.44	1.81	4.08	1.94	3.66
**64**	3.56	5.81	2.68	5.44	3.22	5.12
**65**	4.29	6.66	3.40	6.55	4.12	6.20

**Table 3:** Experimental log *BB* values [43] and molecular descriptors (*A*, *B*, *S*, *E*, *V*) calculated using ACD Percepta for a training set of compounds.

No.	CAS #	*A*	*B*	*S*	*E*	*V*	log *BB*_exp._
1	23830-88-8	0.45	0.90	1.22	1.560	1.5317	0.16
2	21571-08-4	0.45	0.86	1.30	1.690	1.6541	0.47
3	38941-33-2	0.45	0.86	1.54	2.240	1.8119	0.58
4	4205-93-0	0.39	0.90	1.36	1.920	1.6369	0.33
5	40065-09-6	0.45	0.86	1.38	1.870	1.7067	0.41
6	4205-90-7	0.55	1.16	1.34	1.600	1.5317	0.19
7	73590-58-6	0.35	2.05	3.18	2.670	2.5161	−0.82
8	28981-97-7	0	1.55	2.50	2.896	2.2041	−0.04
9	84379-13-5	0	1.55	2.84	2.520	2.7008	−0.09
10	78755-81-4	0	1.50	2.63	1.910	2.0884	−0.29
11	59467-70-8	0	1.38	2.01	2.570	2.2628	0.32
12	99632-94-7	0	1.48	2.52	1.920	2.2773	−0.25
13	2507-81-5	0.75	0.94	1.52	1.906	1.6051	−0.18
14	112598-30-8	0.40	1.69	2.16	2.070	2.0043	−0.66
15	7120-01-6	0.75	0.80	1.00	1.305	1.1382	−0.04
16	104076-38-2	0.40	1.38	2.64	2.689	2.9946	0.14
17	104076-32-6	0.40	1.40	2.69	2.694	2.8898	0.22
18	133099-04-4	0.49	1.58	2.82	2.800	2.2978	−0.62
19	142494-12-0	0.00	1.73	1.83	1.490	2.6577	0.16
20	486-56-6	0.00	1.38	1.49	1.049	1.3867	0.04
21	54-11-5	0.00	1.08	0.92	0.865	1.3710	0.56
22	494-97-3	0.13	0.85	1.02	0.990	1.2301	0.32
23	58-08-2	0.05	1.28	1.72	1.500	1.3632	0.01

**Table 4:** Statistical parameters of cross-validation of MLR models described by Equations (10), (12), (13) and (14); MSE—mean square error, MSEcv*—mean square error of leave-one-out cross validation, MSEcv**—mean square error of leave-50%-out cross validation, PRESS*—predicted residual sum of squares of leave-one-out cross validation, PRESS**—predicted residual sum of squares of leave-50%-out cross validation.

MLR Model	Statistical Parameters	Values
Equation (10)	*N*	23
	*R* ^2^	0.9039
	*Q* ^2^	0.8756
	MSE	0.01784
	PRESS*	0.55198
	MSEcv*	0.01784
Equation (12)	*N*	65
	*R* ^2^	0.8474
	*Q* ^2^	0.8398
	MSE	0.00481
	PRESS*	0.3272
	PRESS**	0.3076
	MSEcv*	0.004799
	MSEcv**	0.004799
Equation (13)	*N*	65
	*R* ^2^	0.8469
	*Q* ^2^	0.8394
	MSE	0.00482
	PRESS*	0.3293
	PRESS**	0.3100
	MSEcv*	0.004841
	MSEcv**	0.004841
Equation (14)	*N*	65
	*R* ^2^	0.8471
	*Q* ^2^	0.8396
	MSE	0.00482
	PRESS*	0.3270
	PRESS**	0.3067
	MSEcv*	0.004817
	MSEcv**	0.004817

**Table 5:** Values of *A*, *B*, *S*, *E*, *V*, topological polar surface area (TPSA), polarizability (*α*), molecular weight (MW) and log *BB* of tested compounds.

Comp. Tested	*A*	*B*	*S*	*E*	*V*	TPSA [A^2^]	*α* [A^3^]	MW [g/mol]	log *BB*_calculated_			
									Equation (10)	Equation (12)	Equation (13)	Equation (14)
**1**	0	1.34	1.73	1.88	1.8144	48.27	27.55	242.28	0.21	0.19	0.20	0.22
**2**	0	1.34	1.67	1.90	1.9553	48.27	29.30	256.30	0.32	0.21	0.22	0.24
**3**	0	1.33	1.81	2.01	1.9368	48.27	29.37	276.72	0.26	0.21	0.22	0.24
**4**	0	1.28	1.80	2.01	1.9368	48.27	29.37	276.72	0.30	0.21	0.22	0.24
**5**	0	1.33	1.84	2.03	1.9368	48.27	29.37	276.72	0.24	0.21	0.22	0.24
**6**	0	1.26	1.91	2.14	2.0592	48.27	31.20	311.17	0.34	0.24	0.24	0.26
**7**	0	1.67	2.13	1.94	2.0297	74.57	30.27	286.29	−0.16	−0.13	−0.11	−0.10
**8**	0	1.67	2.07	1.97	2.1706	74.57	32.02	300.31	−0.04	−0.11	−0.09	−0.08
**9**	0	1.88	2.26	2.01	2.2293	83.80	32.57	316.31	−0.28	−0.23	−0.21	−0.20
**10**	0	1.88	2.26	2.01	2.3702	83.80	34.40	330.34	−0.20	−0.21	−0.19	−0.17
**11**	0	1.65	2.24	2.09	2.1521	74.57	32.09	320.73	−0.12	−0.10	−0.09	−0.07
**12**	0	1.68	2.14	1.94	2.1706	74.57	32.09	300.31	−0.10	−0.11	−0.09	−0.07
**13**	0	1.68	2.08	1.96	2.3115	74.57	33.85	314.38	0.02	−0.09	−0.07	−0.05
**14**	0	1.88	2.24	1.98	2.3702	83.80	34.40	330.34	−0.20	−0.21	−0.20	−0.18
**15**	0	1.62	2.20	2.07	2.2930	74.57	33.92	334.76	0.00	−0.08	−0.07	−0.05
**16**	0	1.66	2.25	2.09	2.2930	74.57	33.92	334.76	−0.05	−0.08	−0.07	−0.05
**17**	0	1.59	2.32	2.20	2.4154	74.57	35.74	369.20	0.05	−0.06	−0.05	−0.03
**18**	0	1.46	2.07	2.24	1.9603	61.41	30.82	280.31	0.06	0.06	0.07	0.09
**19**	0	1.46	2.01	2.26	2.1012	61.41	32.57	294.34	0.17	0.07	0.08	0.10
**20**	0	1.46	2.01	2.26	2.1012	61.41	32.57	294.34	0.17	0.08	0.08	0.11
**21**	0	1.46	1.95	2.29	2.2421	61.41	34.33	308.37	0.29	0.09	0.10	0.12
**22**	0	1.66	2.17	2.28	2.1599	70.64	33.12	310.34	−0.04	−0.05	−0.03	−0.02
**23**	0	1.66	2.19	2.31	2.1599	70.64	33.12	310.34	−0.05	−0.05	−0.03	−0.01
**24**	0	1.45	2.15	2.36	2.0827	61.41	32.64	314.75	0.11	0.07	0.08	0.10
**25**	0	1.40	2.13	2.36	2.0827	61.41	32.64	314.75	0.16	0.08	0.09	0.11
**26**	0	1.45	2.18	2.39	2.0827	61.41	32.64	314.75	0.09	0.08	0.09	0.11
**27**	0	1.38	2.25	2.50	2.2051	61.41	34.47	349.20	0.19	0.10	0.11	0.13
**28**	0	1.38	2.22	2.48	2.2051	61.41	34.47	349.20	0.21	0.09	0.10	0.12
**29**	0	1.42	2.19	2.46	2.1404	48.27	33.92	290.32	0.15	0.26	0.27	0.29
**30**	0	1.43	2.13	2.48	2.2813	48.27	35.68	304.35	0.26	0.27	0.28	0.30
**31**	0	1.43	2.13	2.48	2.2813	48.27	35.68	304.35	0.26	0.28	0.29	0.31
**32**	0	1.43	2.13	2.48	2.2813	48.27	35.68	304.35	0.26	0.28	0.29	0.31
**33**	0	1.63	2.29	2.50	2.3400	57.50	36.23	320.35	0.05	0.15	0.17	0.19
**34**	0	1.63	2.32	2.52	2.3400	57.50	36.23	320.35	0.04	0.16	0.17	0.19
**35**	0	1.63	2.32	2.52	2.4809	57.50	38.05	334.41	0.11	0.18	0.19	0.21
**36**	0	1.43	2.07	2.50	2.4222	48.27	37.43	318.37	0.38	0.29	0.30	0.32
**37**	0	1.41	2.27	2.58	2.2628	48.27	35.75	324.76	0.20	0.27	0.28	0.30
**38**	0	1.37	2.25	2.58	2.2628	48.27	35.75	324.76	0.25	0.28	0.29	0.31
**39**	0	1.41	2.30	2.61	2.2628	48.27	35.75	324.76	0.19	0.28	0.29	0.31
**40**	0	1.34	2.37	2.71	2.3852	48.27	37.57	359.21	0.29	0.30	0.31	0.34
**41**	0	1.43	2.19	2.45	2.2813	48.27	35.75	304.35	0.22	0.28	0.30	0.31
**42**	0	1.42	2.27	2.60	2.4037	48.27	37.57	338.82	0.28	0.30	0.31	0.33
**43**	0	1.42	2.27	2.60	2.4037	48.27	37.57	338.82	0.28	0.30	0.31	0.33
**44**	0	1.42	2.27	2.60	2.4037	48.27	37.57	338.82	0.28	0.30	0.31	0.33
**45**	0	1.43	2.14	2.48	2.4222	48.27	37.50	318.41	0.33	0.30	0.30	0.33
**46**	0	1.43	2.08	2.50	2.5631	48.27	39.26	332.44	0.45	0.31	0.32	0.35
**47**	0	1.43	2.08	2.50	2.5631	48.27	39.26	332.44	0.45	0.31	0.32	0.35
**48**	0	1.43	2.22	2.63	2.5446	48.27	39.26	352.85	0.38	0.31	0.32	0.35
**49**	0	1.43	2.22	2.63	2.5446	48.27	39.33	352.85	0.38	0.31	0.32	0.35
**50**	0	1.43	2.22	2.63	2.5446	48.27	39.33	352.85	0.38	0.31	0.32	0.35
**51**	0	1.63	2.32	2.52	2.6218	57.50	39.88	348.44	0.19	0.19	0.20	0.23
**52**	0	1.64	2.27	2.54	2.9036	57.50	41.63	362.47	0.37	0.21	0.22	0.25
**53**	0	1.63	2.41	2.67	2.7442	57.50	41.70	382.88	0.23	0.21	0.22	0.25
**54**	0	1.63	2.41	2.67	2.7442	57.50	41.70	382.88	0.23	0.21	0.22	0.25
**55**	0	1.63	2.41	2.61	2.7442	57.50	41.70	382.88	0.22	0.21	0.22	0.25
**56**	0	1.43	2.22	2.63	2.5446	48.27	39.33	352.85	0.38	0.31	0.32	0.34
**57**	0	1.41	2.30	2.60	2.4037	48.27	37.57	338.82	0.27	0.29	0.31	0.33
**58**	0	1.41	2.38	2.75	2.5261	48.27	39.40	373.27	0.31	0.31	0.33	0.35
**59**	0	1.41	2.38	2.75	2.5261	48.27	39.40	373.27	0.31	0.31	0.33	0.35
**60**	0	1.41	2.38	2.75	2.5261	48.27	39.40	373.27	0.31	0.32	0.33	0.35
**61**	0	1.43	2.20	2.45	2.4222	48.27	37.58	318.37	0.29	0.29	0.30	0.32
**62**	0	1.43	2.14	2.48	2.5631	48.27	39.33	332.40	0.41	0.31	0.32	0.34
**63**	0	1.42	2.28	2.58	2.5446	48.27	39.40	352.82	0.34	0.30	0.32	0.34
**64**	0	1.42	2.31	2.60	2.5446	48.27	39.40	352.82	0.33	0.31	0.32	0.35
**65**	0	1.35	2.38	2.71	2.6670	48.27	41.22	387.26	0.43	0.34	0.35	0.37
